# Social Comparison on Social Media Increases Career Frustration: A Focus on the Mitigating Effect of Companionship

**DOI:** 10.3389/fpsyg.2021.720960

**Published:** 2021-10-15

**Authors:** Nao Fukubayashi, Kei Fuji

**Affiliations:** ^1^R&D Center for Working Persons’ Psychological Support, University of Tsukuba, Tsukuba, Japan; ^2^Faculty of Human Sciences, R&D Center for Working Persons’ Psychological Support, University of Tsukuba, Tsukuba, Japan

**Keywords:** social comparison, career frustration, companionship, social media, SNS, experience sampling method (ESM), multilevel SEM

## Abstract

Social media has become a platform for the daily exchange of information. Although some studies have explored the role and influence of social media on career development, few have examined how daily social media use impacts individuals’ perceptions and emotions regarding their careers. The present study examined this issue using two surveys. We predicted that social comparison would mediate the link between social media usage and its psychological impact. Moreover, we hypothesized that the impact would be mitigated by social interactions (companionship). Study 1 (a self-reported survey that included 309 Japanese employees) demonstrated that viewing other users’ positive posts about their careers could lead to career frustration through social comparison. Concurrently, this study revealed that daily casual interaction with others reduced career frustration. Study 2 was based on an analysis of 1,254 responses obtained from a 7-day experience sampling method survey. It revealed that viewing other people’s career-related posts was associated with upward, downward, and non-directional social comparison. In turn, upward social comparison evoked career frustration at both between- and within-person levels, while downward comparison decreased career frustration at a between-person level. Similar to Study 1, the results of Study 2 indicated that career frustration was mitigated by casual communication with others. Both studies provide evidence that (1) daily social media use affects one’s perception and feelings about their career through social comparison, and (2) career frustration evoked through virtual interactions with others is mitigated by casual interactions in a face-to-face setting.

## Introduction

The nature and environment of careers have dramatically changed over the past several decades (e.g., Arthur, 1994; Hall, 2004; Sullivan and Baruch, 2009). With the advent of the Internet and social media, individuals can actively gather career-related information on their own. For example, LinkedIn, a business-focused social networking site, boasts 756 million users in 200 countries (LinkedIn, 2021), and its use is also expanding in Japan. Utz and Breuer (2016) and Davis et al. (2020) have demonstrated that the use of social media (LinkedIn) can benefit individuals’ career development in various aspects, such as information gathering and networking. However, there is still hope that further academic research on the use and potential of social media in career development will be performed (Sullivan and Al Ariss, 2021). Moreover, considering that social media has gained considerable popularity, to the extent that people can remain in contact on a daily basis, it is possible for individuals to obtain career-related information through social media posts, even when they do not intend to actively collect such information. Regardless of the individual’s intention, such information about careers on social media could have an impact on the viewer’s career. Therefore, in this study, we examined the effects of daily social media browsing on an individual’s career.

## Theoretical Framework

### Career-Related Social Comparison on Social Media and Career Frustration

Since the 2010s, research has investigated the positive impacts of social media use, such as enhancing well-being (Rae and Lonborg, 2015; Burke and Kraut, 2016) and leading to an increased sense of belonging and life satisfaction (Oh et al., 2014). However, there is also a growing interest in how social media negatively affects depression and emotional states. O’Keeffe and Clarke-Pearson (2011) examined the effects of social media use on children and adolescents and their families, and reported that adolescents who used Facebook for extended periods displayed depressive symptoms, which they named “Facebook depression.” Since then, a number of studies have examined the negative psychological impact of social media (for a review, see Seabrook et al., 2016).

Among these studies, there has been a growing interest in the mechanisms underlying the ability of social media to cause negative emotional states. The environmental characteristics of social media, in which users tend to portray a more positive and idealistic self-image than reality through photos and posts, have drawn significant attention (Zhao et al., 2008; Nadkarni and Hofmann, 2012; Qiu et al., 2012). Previous studies have noted that such environmental characteristics could have a negative impact on users’ psychological states. For example, Stronge et al. (2015) found that social media diminished the user’s sense of belonging through passive consumption of positive information about others. By adopting the concept of the availability heuristic, Chou and Edge (2012) established that, as the positive information presented on social media was more effortlessly recalled than reality, people were more likely to conclude that others were happier and had better lives than their own based on others’ self-presenting information on social media. The authors also demonstrated that, due to correspondence bias, which caused people to conflate the positive content on social media with other people’s personalities and characteristics, people tended to ignore the background situations and believed other people’s lives were better than their own overall.

One of the principle explanations for why such an environment could have a negative psychological impact on users is social comparison (Festinger, 1954). Social comparison is the process of assessing one’s position and ability in society by making comparisons with others, and is classified into three types according to its direction, as follows: upward comparison, where people perceive others to be superior to themselves; downward comparison, where people believe that they are superior to others; and non-directional comparison, where people compare themselves with others in neither direction. Considering that social comparison is more likely to occur when the other person is similar to oneself in terms of the comparison domain of interest and traits, social media, where users are connected with familiar people, offers a fertile environment for social comparison (Vogel and Rose, 2016). Vogel et al. (2014) predicted that comparisons on social media with the aforementioned environmental characteristics are biased toward upward comparisons, and found that individuals who used social media more frequently tended to have lower self-esteem *via* social comparison. A meta-analysis found that the social comparison was more strongly associated with depression than time spent on and frequency of checking social media (Yoon et al., 2019). A recent review reported that social comparison had harmful consequences when people viewed other users’ posts and information (other-focused or passive use) rather than posting about themselves or viewing their own profiles (self-focused or active use; Vogel and Rose, 2016; Verduyn et al., 2017).

While the role and impact of social media on careers have not yet been established, some studies on social comparison in social media have examined the aspect of careers. In their qualitative research, Pisarik et al. (2017) found that social media served as a platform for students to compare their situation to that of other students who were starting a job or building outstanding careers, which worsened their anxiety. In their interviews and experimental study, Haferkamp and Krämer (2011) revealed that men perceived a greater gap between their ideal and real careers when they saw more successful career profiles on social media than less successful career profiles compared to their own.

Based on the research on social media-induced social comparison and career-related comparisons, we made the following hypothesis:

[H1] Daily exposure to others’ posts about careers increases anxiety and frustrations about one’s own careers *via* social comparison.

### Companionship as a Buffer Against Negative Psychological States

While many studies have demonstrated the associations between daily social media use, social comparison, and negative psychological outcomes, such as depression and lower self-esteem, few have examined the factors that reduce these negative outcomes. In this regard, it would be meaningful to identify factors that might counteract the negative psychological outcomes of social comparison on social media.

In this study, we focused on “companionship” as a potential factor that might alleviate career frustration and anxiety about careers that are associated with daily social media exposure. Companionship is defined as “shared leisure and other activities that are undertaken primarily for the intrinsic goal of enjoyment” (Rook, 1987). In a broad sense, companionship is sometimes considered as a type of social support; however, these concepts can be differentiated, in that companionship focuses on mutual enjoyment and entertainment, whereas social support focuses on problem alleviation and stress reduction.

In a series of studies with college students and local communities, Rook (1987) demonstrated that social support and companionship had different effects on stress and mental health. Namely, when confronted with seriously stressful events, social support was effective in reducing the symptoms of stress; however, for daily stressful events, social support was less effective, and companionship was significantly effective in both reducing stress symptoms and promoting mental health (Rook, 1987; Bolger and Eckenrode, 1991). Newsom et al. (2005) found that, among various types of positive social exchanges, including instrumental and emotional support, companionship (meaning day-to-day enjoyment) was appraised more positively and most strongly predicted greater well-being and less distress. A study in the workplace also revealed similar results, whereby social interactions, such as “having a pleasant conversation” or “laughing together” with a colleague or supervisor, suppressed negative emotions about stressful events (Buunk and Verhoeven, 1991). Moreover, in Japan, everyday casual interactions have been shown to reduce stress reactions in the workplace (Kishimoto and Fuji, 2020).

Given the existing evidence that companionship can mitigate daily stress, companionship could also reduce social media-induced stress, which was the focus of the present study. Although the effects of companionship on career-related stress is not yet known, the research on anxiety reduction (Conwell et al., 2021) and work-related stress reduction means that it is conceivable that companionship alleviates anxiety and negative emotional states about one’s career. This led us to the second hypothesis, as follows:

[H2] Casual interactions with others eases the negative psychological impact of social comparison on social media.

### Experience Sampling Method

In this study, we adopted the experience sampling method (ESM or “ecological momentary assessment”; Stone and Shiffman, 2002) to capture daily social media use, the occurrence of social comparison, companionship, and their influences on users’ psychological states.

The ESM is considered to be an effective approach to understand the relationship between the environment and a person’s cognitive and affective state. Measurements are taken several times per day over a certain period in participants’ daily lives (Csikszentmihalyi et al., 1977; Prescott et al., 1981). The relatively recent development of portable information devices, such as smartphones, means that the ESM has been used with increasing frequency, and has been demonstrated to be an effective method for capturing the everyday psychological changes that may be overlooked in retrospective assessments (Hofmann et al., 2012; Hofmann and Patel, 2015).

This methodological trend has also been seen in social media-related research (e.g., Verduyn et al., 2015; Fardouly et al., 2017). For example, using an ESM survey that involved sending five text messages a day to participants for 2 weeks, Kross et al. (2013) found that using Facebook undermined affective and cognitive well-being. This ESM intensive longitudinal survey also allowed the researchers to reject the possibility that other direct interactions (i.e., other than those on Facebook) undermined well-being, or that the findings were the result of a reverse pathway, whereby mood or depression predicted Facebook use.

For social comparison, which is a relatively automatic response (Gilbert et al., 1995), the ESM or daily diaries (only recorded once per day for a certain period; Arigo et al., 2020) have also been utilized to avoid recall bias. These methodologies have become increasingly popular, particularly since 2011, in studies on social comparison on social media platforms (Arigo et al., 2020). Using the daily diary method, Steers et al. (2014) revealed that, on days when people spent more time on Facebook, they engaged in upward and non-directional social comparisons that, in turn, increased their daily depressive symptoms.

The same methodologies have been used to measure daily social interactions (Kross et al., 2013; Verduyn et al., 2015). For example, Ram et al. (2014) used smartphones and a web-based survey to employ an ESM protocol in their study on daily casual interactions and affective changes.

Given these previous findings, we adopted the ESM to examine the associations between career-related social comparison in daily social media use and companionship, as well as the associated psychological changes. We examined our two hypotheses using not only a self-reported recall (Study 1) but also the ESM (Study 2).

## Study 1

### Materials and Methods

#### Participants and Procedures

Participants in Study 1 were recruited using a Japanese marketing research company. Before completing the response pages, participants were informed of the study purpose and the estimated completion time. They were also provided with notes on the study ethics, which stressed that the survey was anonymous and voluntary, and that they could drop out whenever they desired. Considering the user distribution of social media in Japan, Facebook users in their 20s–40s were targeted as participants. In addition, participants were also screened according to their number of working years (three or more) and contract type (excluding part-time and temporary workers). Out of 405 potential participants, 309 (197 males, 105 females, and 7 who did not report their gender) completed the survey, ranging from 26 to 49 years old [mean (M) = 39.43 years, standard deviation (SD) = 6.66 years]. Each participant received compensation (approximately 20 JPY) from the research company. Before the start of the study, ethical approval was obtained from the Human Research Ethics Committee of the University of Tsukuba.

#### Measures

##### Viewing Other Users’ Career-Related Posts on Social Media

Nine items were used to measure the frequency of viewing other users’ career-related posts on social media. The items were preceded by the following statements: “How frequently do you view other users’ work- and career-related posts on Facebook?,” “viewing other users’ posts on their achievements in their work or career (current/so far),” “viewing other users’ posts on their successful experiences in their work or career (current/so far),” “viewing other users’ posts on their satisfying working lives (current/so far),” “viewing other users’ posts on the expectations around their future careers,” “viewing other users’ posts on the goals for their future careers,” and “viewing other users’ posts around what they want to pursue in their future careers.” Each item was rated using a 5-point Likert scale that ranged from “never” to “very often.”

##### Career-Related Social Comparison

Career-related social comparison was measured using the career version of the two-factor Iowa-Netherlands Comparison Orientation Measure, which was developed by Gibbons and Buunk (1999).

We completed a translation and adjustment process to measure career-related social comparison among workers. First, with the approval of Prof. Frederick X. Gibbons, we translated the original scale into Japanese following the instructions from The Professional Society for Health Economics and Outcomes Research Task Force for Translation and Cultural Adaptation report (Wild et al., 2005). Thereafter, we adjusted the wording of each item on the career-related comparison. In addition, to focus on career comparison on social networking sites, in the survey instructions, we asked respondents to provide the degree to which they agreed with each item related to their behaviors on social networking sites.

Before the main analysis, we conducted a preliminary survey with 75 workers to confirm the understandability of the expressions used for each item, using a one-sample *t*-test to the median. The result confirmed that none of the items had an understandability that was significantly below the median; thus, we decided to use the scale for further analyses.

##### Career Frustration

Career frustration was measured using the Feeling under Pressure subscale of the 16-item scale created by Ono and Okada (2014). The reliability, internal validity, and criterion-related validity for the scale were reviewed. The following items were included in the subscale: “I feel stuck with no way out,” “I feel I have reached a stalemate and cannot do anything about it,” “It takes so long to achieve my goal that I cannot stand it,” “I want to escape to somewhere else,” “I feel that I am at a dead end,” and “No matter what, I must get out of my current situation.” Each item was rated using a 5-point Likert scale that ranged from “agree” to “disagree.”

##### Companionship

To measure companionship, we extracted items used by Rook (1987) and Buunk and Verhoeven (1991). These items asked respondents to report how many times they had had direct social interactions with others in the past 3 months, such as dining out or going to the movies. Respondents were also asked to rate how pleasant the interactions were using a 5-point Likert scale that ranged from “not at all pleasant” to “very pleasant.”

#### Data Analysis

To assess the structural model, we conducted a covariance structure analysis using the full information maximum likelihood method with SPSS Amos version 26.0 (IBM Corp., Armonk, NY, United States). We used the chi-square value divided by the degrees of freedom (χ^2^/df), comparative fit index (CFI), and the root mean square error of approximation (RMSEA) to assess the structural model fit. For the CFI, values that were ≥0.90 were considered to indicate an acceptable fit (Marsh and Hau, 1996; Salisbury et al., 2002). We referred to MacCallum et al. (1996) for the RMSEA, whereby values in the range of 0.05–0.08 are defined as a fair fit and those in the range of 0.08–0.10 as a mediocre fit.

### Results

#### Descriptive Statistics and Pearson’s Correlations

The descriptive statistics and the Pearson’s correlations between the variables of interest are shown in Table 1.

**TABLE 1:** Cronbach’s alpha, means, standard deviations, and correlation coefficients for the study variables (*N* = 309).

		α	M	SD	2	3	4	5	6
(1)	Viewing other users’ career-related posts on social media	0.98	1.71	0.90	0.68**	0.68**	0.03	–0.09	0.19**
(2)	Ability to drive their own career	0.95	1.82	0.90		0.86**	–0.04	–0.10	0.26**
(3)	Opinions on career development	0.94	1.94	0.98			0.00	–0.05	0.22**
(4)	Number of interactions	–	3.55	5.47				0.24**	–0.06
(5)	Pleasantness of interactions	–	3.73	1.02					–0.22**
(6)	Career frustration	0.93	2.65	0.93					

**p < 0.01.

*α, Cronbach’s alpha; M, mean; SD, standard deviation.*

In addition to the variables shown in Table 1, we conducted an additional analysis with age and gender to confirm their impact on each variable. Based on the result of the correlation analysis with age (the coefficients with social media viewing and social comparison were significant but small), and of the *t*-test with gender (no significant result), we decided to exclude these variables in the subsequent analysis.

#### Measurement Model—Social Comparison

Before analyzing the structural model, we conducted a confirmatory factor analysis for the social comparison measurement model using SPSS Amos version 26.0 (IBM Corp.). The χ^2^ and CFI suggested a good fit of the model [χ^2^(43) = 197.965 (*p* < 0.01), CFI = 0.953]; however, the RMSEA was slightly elevated, at 0.108. We decided to adjust the model by excluding items 5 and 11 for the following reasons. First, the factor loading of these two items was low (below 0.40) compared to the other items. Second, in the original scale by Gibbons and Buunk (1999), each factor was comprised of the items that had over a 0.50 factor loading. Third, previous studies have not reached a consensus on how to deal with these two reverse-coded items. Gibbons and Buunk (1999) included items 5 and 11 in the ability and opinion factors, respectively. Contrastingly, Schneider and Schupp (2014) found that item 11 had a higher factor loading in the ability factor, while its absolute value stayed below 0.40; thus, they suggested the possibility of relocating item 11 to the ability factor or excluding it entirely, which significantly improved the goodness of fit. Moreover, according to Toyama (2002), who attempted to develop a Japanese version of the social comparison scale, item 11 again presented a higher factor loading on the ability factor. After excluding items 5 and 11, the confirmatory factor analysis was repeated. The results were as follows: χ^2^(26) = 105.040 (*p* < 0.01), CFI = 0.975, and RMSEA = 0.099. Although the RMSEA was slightly elevated, given the suggestion by MacCallum et al. (1996) that an RMSEA that ranged from 0.08 to 0.10 indicated mediocre fit, we decided to apply this model to our further analyses.

Following the original scale labels, we labeled the two factors as “ability to drive one’s own career” (5 items) and “opinions on career development” (4 items). Each item was rated using a 5-point Likert scale that ranged from 1 (strongly disagree) to 5 (strongly agree).

#### Structural Model Analysis

##### Goodness of Fit

The structural modeling was performed using SPSS Amos 26.0 (IBM Corp.). The structural model yielded an acceptable fit: χ^2^ (429) = 1169.502 (*p* < 0.01), CFI = 0.93, and RMSEA = 0.075.

##### Results Relating to the Hypotheses

**FIGURE 1:**
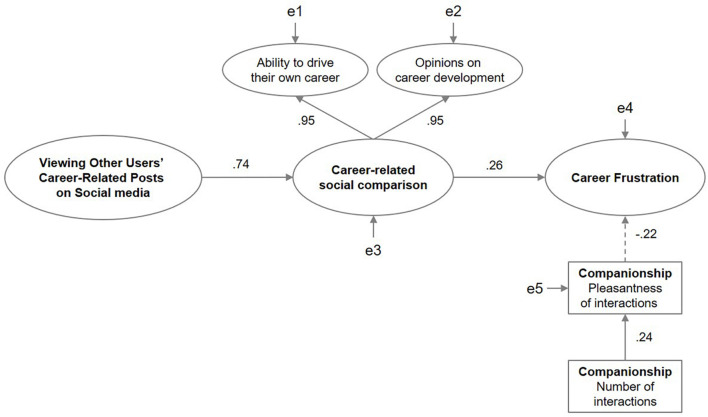
Structural relationships between the studied variables: viewing other users’ careers on social media, career-related social comparison, career frustration, and companionship. The observed variables are indicated by rectangles and the latent variables are indicated by ellipses. Positive coefficients are shown using solid arrows, while negative coefficients are shown using dotted arrows. All paths are significant at *p* < 0.001. All coefficients are standardized.

The structural model is represented in Figure 1. Viewing other users’ careers on social media showed a strong positive association with career-related social comparison (β = 0.70, *p* < 0.001), and this, in turn, predicted career frustration (β = 0.27, *p* < 0.001). Contrastingly, the number of direct interactions had a positive effect on the pleasantness of interactions (β = 0.24, *p* < 001), and this eventually decreased career frustration (β = −0.21, *p* < 0.001).

### Discussion

In Study 1, we first tested H1, which predicted that career-related social comparison would be evoked by viewing other users’ careers on social media, and would in turn induce career frustration. Our results suggested that viewing how others were building careers through their career-related posts on social media led respondents to compare their own careers with other users’ careers. Moreover, this comparison eventually brought about career frustration and the feeling of pressure to get out of their current occupational situation. Based on these findings, H1 was supported. These findings are also in accordance with those of previous studies, whereby passive social media usage was found to cause negative psychological feelings *via* social comparison (Vogel et al., 2014; Stronge et al., 2015).

In contrast, companionship, that is, off-line interactions, mitigated career frustration. Companionship was considered to reduce minor stressors or daily stress. Since social comparison can be regarded as daily stress, the results of this study correspond with previous research on companionship (Rook, 1987; Buunk and Verhoeven, 1991). Based on these findings, H2 was also supported.

## Study 2

Study 2 investigated whether Study 1’s model was observable on a daily basis. While Study 1 explored the individual differences regarding career-related social comparison, Study 2 expanded on the within-person level (daily activity) and compared the tendencies at the within- and between-person levels. To gather further information on social comparison, we considered the directions of social comparison in Study 2. The study used a 7-day ESM to capture the participants’ daily behaviors and their psychological impacts.

### Materials and Methods

#### Participants

Study 2 participants were recruited from the Study 1 participants. At the end of the Study 1 questionnaire, we explained the purpose and the outline of Study 2, and that it was being conducted over a three-month interval. Respondents who were willing to participate were asked to provide their email addresses so that they could receive and answer the survey during the day. The candidates were also informed that they could withdraw from the study at any time. To adjust the timing of the questionnaire notifications, we also asked participants to provide their lunch timings and the approximate times at which they finished work. In total, 166 people agreed to participate.

Each participant received compensation (approximately 20 JPY) from the research company for responding to each questionnaire. Before the study began, ethical approval was obtained from the Human Research Ethics Committee of the University of Tsukuba.

#### Procedures

Three months after the completion of Study 1, an advance notice with details of the study was distributed and participants were asked to indicate whether they wished to withdraw before starting the study. A few days later, participants received emails with a participation link twice a day for 7 consecutive days. The advance notice and each email stated that they could withdraw from the study at any point. The first questionnaire was sent approximately at lunchtime, between 11:45 and 14:00, and the second questionnaire was sent between 21:00 and 22:30. The link redirected participants to an online survey where they were asked to report their activities related to social media viewing, social comparison, and companionship during the preceding 3 hours, as well as their psychological state regarding career frustration at the time. The email also stated that participants could answer the questionnaire at any time, as long as there were 3-hour or longer intervals between the two daily questionnaire responses to avoid data duplication.

#### Measures

##### Viewing Other Users’ Career-Related Posts on Social Media

For viewing other users’ career-related posts on social media, we asked respondents about their activity over the past 3 hours regarding viewing other users’ posts using six items that were extracted from Study 1. These items included “viewing other users’ posts on their successful experiences in their work or career (current/so far)” and “viewing other users’ posts on their future career goals.” Each item was rated using a 6-point Likert scale that ranged from “never” to “very often.”

##### Career-Related Social Comparison

In reference to the work by Steers et al. (2014), six items from the career version of the Iowa-Netherlands Comparison Orientation Measure used in Study 1 were adapted to measure non-directional, downward, and upward social comparisons (two items for each). These items included “I paid a lot of attention to how I have built my career compared with how other people have built their careers,” “I felt less confident about the career I have developed compared to other people,” and “I believe that I have accomplished a better career than other people have.” Participants were asked to respond according to their activities in the preceding 3 hours. All items were rated using a 9-point Likert scale that ranged from “strongly disagree” to “strongly agree.”

##### Companionship

Companionship was assessed according to the lengths of time that participants spent chatting or in conversations unrelated to work, as well as the pleasantness of those interactions. We also asked who they had interactions with. The lengths of time were measured according to 19 levels that were divided into 10-min segments ranging from “0–10 min” to “2 h 51 min–3 h.” Given that Study 2 focused on the participants’ daily activities, we adjusted the number of direct social interactions in Study 1 to the length of time dedicated to chatting in the preceding 3 hours. Pleasantness was rated using a 5-point scale that ranged from “not at all pleasant” to “very pleasant.”

##### Career Frustration

The three highest factor loading items in Study 1 were selected from the career frustration scale (Ono and Okada, 2014) to measure career frustration. Each item was measured using a 5-point Likert scale that ranged from “agree” to “disagree.”

#### Data Analysis

Throughout the 7-day investigation, 143 participants completed 1,394 questionnaires. Data that met the following criteria were excluded from the analysis: (1) responses with five or more items missing (one-third of the total items), (2) responses that did not follow the response timing rule, and (3) entire responses from individuals who completed less than five questionnaires. Ultimately, 1,254 answers comprising 90% of the total 1,394 answers from 111 participants (68 males, 40 females, and 3 who did not provide information about their gender; M age = 39.11 years; *SD* = 6.85 years; age range, 26–49 years) were included in the analysis.

We predicted that social comparison would be induced by each exposure to social media, and that there would also be individual differences in the influence of social media viewing on social comparison. Thus, the data were analyzed based on a two-level structure (i.e., activities nested within persons). MPlus 8.5 (Muthén and Muthén, 2021) was used to create the multilevel structural equation models to analyze the structures at the within- and between-person levels. Missing data were handled using the full information maximum likelihood and the maximum likelihood estimation with robust standard errors as estimators. Regarding the indices of the model fit, 0.08 was the cut-off value for the standardized root mean residual (Hu and Bentler, 1999), and the same standards as the single level models were followed for the RMSEA and CFI.

### Results

#### Descriptive Statistics and Intraclass Correlations

The descriptive statistics of the variables of interest and intraclass correlation (ICC) results are provided in Table 2. The ICC indicated the relative amount of variance that was attributed to individual differences or differences by situations. The ICCs for social media viewing and social comparisons were 0.58 and 0.52–0.55, respectively, which suggests that the total variance was almost equally distributed between the within- and between-person levels. For companionship, the ICCs for both the length of time and pleasantness were just below 0.40, which suggests that approximately 40% of the variance for companionship was attributable to individual differences, whereas 60% was attributable to situational differences. In contrast, the ICC for career frustration was 0.80, which suggests that most of the variability in career frustration was interpersonal.

**TABLE 2:** Descriptive statistics and intraclass correlation coefficients for the variables of Study 2.

	n	M	SD	Min	Max	ICC
Viewing other users’ career- related posts on social media	1,254	1.50	1.00	1	6	0.58
Upward comparison	1,254	2.45	2.02	1	9	0.52
Downward comparison	1,254	2.52	2.00	1	9	0.54
Non-directional comparison	1,254	2.44	1.96	1	9	0.55
Length of time of interactions	1,239	3.76	3.75	1	19	0.38
Pleasantness of interactions	1,088	3.81	1.45	1	6	0.37
Career frustration	1,253	2.30	1.24	1	5	0.80

*n, number; M, mean; SD, standard deviation; Min, minimum; Max, maximum; ICC, intraclass correlation coefficients.*

Pertaining to companionship, we categorized relationships with whom the participants had interactions into four categories, based on 870 valid responses out of 1,254 total responses. Considering that each respondent interacted with more than one person, we counted the types of relationship for each respondent, as follows: (1) colleagues and professional contacts (43.1%), (2) family and relatives (35.1%), (3) friends, acquaintances, partners (18%), and (4) shop staff, etc. (3.8%).

#### Two-Level Structural Model

**FIGURE 2:**
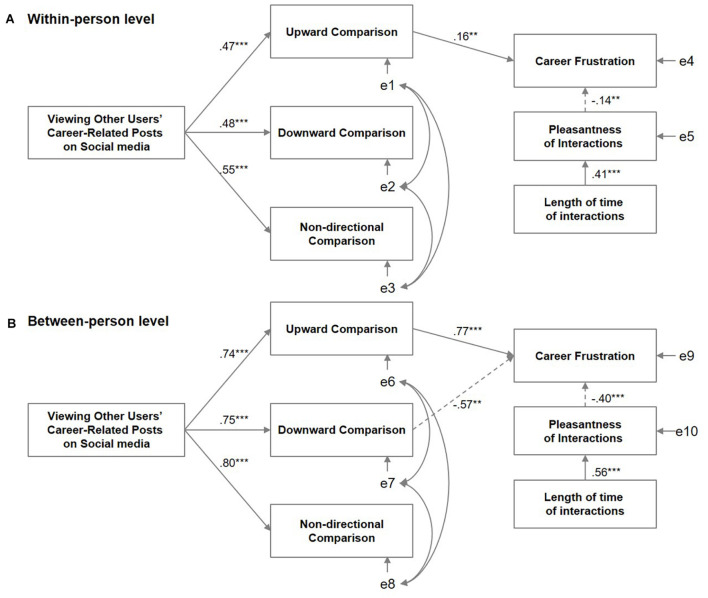
The two-level structural equation model for viewing other users’ careers on social media, career-related social comparison, career frustration, and companionship. **(A)** Within-person level. **(B)** Between-person level. Non-significant paths were removed for the sake of clarity. Statistical significance is indicated by ***p < 0.001, **p < 0.01. Standardized coefficients are shown. Positive coefficients are shown using solid arrows, while negative coefficients are shown using dotted arrows. The factor correlations among the social comparison factors are: upward − downward = 0.60 (within-person)/0.62 (between-person), upward − non-directional = 0.75 (within-person)/0.80 (between-person), downward − non-directional = 0.73 (within-person)/0.84 (between-person).

Figure 2 presents the results of the multilevel structural equation models for the variables of interest. Only statistically significant paths are shown and the coefficient values are standardized. The model results were as follows: RMSEA = 0.034, standardized root mean residual (within-person) = 0.036, standardized root mean residual (between-person) = 0.031, and CFI = 0.98.

Overall, the results at both within- and between-person levels demonstrated that career frustration was affected by social media viewing *via* social comparison, and companionship reduced the career frustration caused by social media viewing and social comparison. Closer inspection of the details for each level established that, at the situational level, viewing other users’ career-related posts on social media predicted social comparison regardless of the direction (upward β = 0.47, downward β = 0.48, non-directional β = 0.55). Whether social comparison consequently increased career frustration depended upon its direction. Career frustration was only evoked when people felt a sense of inferiority or felt less confident through upward social comparison (β = 0.16). The results also indicated that the length of time of companionship increased the pleasantness of interactions (β = 0.41), which, in turn, reduced career frustration (β = −0.14).

Similar results were found at the between-person level; however, there were some additional findings. Participants who viewed other users’ career-related posts on social media were more likely to engage in social comparison in all directions (upward β = 0.74, downward β = 0.75, non-directional = β = 0.80). As with the results at the within-person level, an upward comparison was a strong predictor for career frustration (β = 0.77). Moreover, an interesting contrast to the within-person level was noted; at the between-person level, downward social comparison reduced career frustration (β = −0.57). This finding may indicate that those who were more likely to view other users’ positive career stories had feelings of superiority about their own careers in comparison with those of other users. In terms of companionship, people who were more likely to have longer interactions with others had higher chances of having a pleasant time compared to their counterparts (β = 0.56) and, in turn, felt less pressure regarding their careers (β = −0.40).

### Discussion

To summarize, Study 2 mostly supported our hypotheses. There were some additional findings to Study 1, whereby the three directions of social comparison (upward, downward, and non-directional) had different effects on career frustration.

Overall, using cross-sectional self-report and diary methods, Studies 1 and 2 revealed that viewing other users’ career-related social media posts was associated with a career-related social comparison that, in turn, increased frustration about the participant’s career. They also indicated that companionship acted as a buffer to these career-related negative emotional states.

Study 2, which was conducted using the ESM, revealed that viewing other users’ career-related stories on social media was significantly associated with social comparison at each exposure. Moreover, Study 2 found that viewing other users’ career stories almost equally caused all types of social comparison. These results correspond with previous findings that support the idea that social media use causes social comparison (Chou and Edge, 2012; Nisar et al., 2019). Interestingly, however, Study 2’s findings that social media was an equally strong positive predictor for downward and upward social comparison contradict the results from Steers et al. (2014) and Vogel et al. (2014). This could be attributed to the focus of the present study on “career.” When the posts were career-specific, the viewers would be able to set clearer standards of comparison in terms of, for example, income, economic and social status of the occupation, accomplishments that have been achieved, and the excellence of the work, as compared to their general daily lives. The social comparisons could result in the feeling that “they have a better career than me” (upward); however, the conclusion that “their career is not that great” (downward) could be drawn with equal likelihood.

Moreover, both studies revealed that the social comparison evoked by viewing social media affected career frustration. These results are consistent with those of several studies that examined the negative psychological outcomes of social media use and social comparison (Feinstein et al., 2013; Lee, 2014). Study 2 expanded on the findings of Study 1, and established that different types of social comparison had unique effects on career frustration. Specifically, at the within-person level, only upward social comparison increased career frustration; at the between-person level, upward social comparison had a similar but stronger effect on career frustration, while a downward social comparison had the opposite effect. This effect of downward social comparison has been noted elsewhere, whereby downward social comparison reportedly improved psychological state (Johnson and Knobloch-Westerwick, 2014). To summarize this study, while the positive relationship between upward social comparison and career frustration indicated deterioration of a person’s perception of their career, making a downward social comparison may have provided them with feelings of relief or comfort by reinforcing the idea that their career was better than others’ on social media. Given these results, one of the reasons that social media continues to attract people could be its use as a tool for providing people with pleasant feelings in the form of a sense of superiority or relief, and not solely with more unpleasant feelings, such as frustration.

Regarding career frustration, although the major variance was thought to be attributable to the individual differences based on the high ICC value, the within-person level results indicated that each exposure to social media was also positively related to upward social comparison.

Finally, Studies 1 and 2 demonstrated that social interactions could contribute to decreased career frustration. Considering that career-related frustration could be considered as daily stress, as opposed to serious stress, these results are in accordance with previous findings that companionship reduces minor stresses (Rook, 1987; Buunk and Verhoeven, 1991; Kishimoto and Fuji, 2020). These findings not only suggest that daily stress derived from comparative interactions in social media can be mitigated by in-person interactions, but they also indicate that social interaction can help reduce anxiety and stress regarding the unknown future with respect to one’s career. To note, an alternative explanation for this decreased career frustration through interactions with people in domains unrelated to one’s career or work was also considered; as proposed by theories such as self-complexity (Linville, 1985, 1987), possessing more distinct self-aspects may have reduced the negative impact of social comparison. However, in the present study, participants in Study 2 communicated more with colleagues in the workplace and professional contacts than with family and friends, who are usually distant from one’s work and career. We therefore explained the result by the buffering effect of day-to-day companionship rather than the alternative explanation.

This study has several limitations. First, we only examined the amount of viewing of career-related posts. However, it is possible that exposure to other types of posts, such as cooking, hobbies, and travel posts, may also induce social comparison. Future work could therefore consider the relative amount of viewing of career-related posts to a wider range of topics to examine the impact of viewed content more closely.

Considering the different purposes of social media, the different types of “friend lists,” and the nature of the relationship, such as whether it is more business-related or a casual friendship, may yield different results. Previous studies have verified that different types of social media and the degree of closeness between the target and the perceiver affect the occurrence of social comparison (Vogel and Rose, 2016; Chae, 2018). This indicates that the occurrence of social comparison may vary according to type of information, and from whom, users are exposed to. The purposes and types of networks on social media are the keys to such variation in information; thus, future research should take these issues into account.

Previous research on related topics, such as Facebook Depression, has mostly agreed that passive viewing and social comparison have negative effects. Based on these findings, the present study focused on these aspects. However, Meier and Schäfer (2018) have reported that non-directional social comparison in social media can have positive outcomes, such as inspiration. In addition, the outcome of passive viewing may vary depending on mediators, such as self-esteem (Chen et al., 2016). Thus, future work could incorporate such positive outcome factors and mediators. Similarly, it has also been shown that differences in personality, such as neuroticism, may affect the evocation of social comparison and the level of affective consequences (VanderZee et al., 1996), which could indicate that differences in personality influence the mitigating effect of companionship found in the present study. Therefore, taking personality factors into account may also give new insights in future work.

Regarding the effect of companionship on reducing career frustration, these findings could simply be the result of a tradeoff between the time spent on social media and companionship. That is, spending more time on companionship reduces the amount of time spent on social media and this, in turn, reduces the negative impact of social comparison on career frustration. We conducted additional analyses to consider this point, and found (1) no negative correlation between the length of time of companionship and social media viewing, and (2) that the length of time of companionship did not significantly affect career frustration.

This study did not take income or social status into account. Here, we modeled the intangible aspects of a career (such as business success, achievement, and future career goals) to induce social comparison. We did not include the tangible aspects, such as income and social status; however, these differences could also induce social comparison; therefore, future research should also consider these aspects.

This study examined the associations between social media use, career-related social comparison, and career frustration with respect to interpersonal and intrapersonal differences. The results of this study contribute to the existing knowledge about people’s social media-related behaviors and their psychological impact. Notably, within the limited number of studies on the influence of social media on careers, the present study is meaningful because it demonstrated the possibility that daily social media use has an impact on people’s perception of their careers. This has been particularly interesting in recent years, during which social media has become a popular platform for daily information exchange and people’s perceptions of their work and careers have varied significantly. We hope that future studies will continue to add to the body of work in this area of interest.

To date, studies have paid much attention to the mechanisms underlying the impact of social media use, and personality traits have been proposed as major mediators or moderators of the psychological impacts (Feinstein et al., 2013; Rae and Lonborg, 2015; Appel et al., 2016; Wang et al., 2017). However, we illustrated the possibility that behavioral-level factors, such as social interaction, could mitigate the negative psychological impacts of social media use and social comparison. This could be instrumental in developing new interventions to manage the effects of social media. From 2020, a significant amount of communication has moved online and a major transformation of communication in the workplace is anticipated. In such situations, we may easily miss out on casual communication; the current study indicates that we should place greater importance on such daily interactions.

## Data Availability Statement

The raw data supporting the conclusions of this article will be made available by the authors, without undue reservation.

## Ethics Statement

The studies involving human participants were reviewed and approved by the Human Research Ethics Committee of the University of Tsukuba. Written informed consent for participation was not required for this study in accordance with the national legislation and the institutional requirements.

## Author Contributions

NF and KF contributed to the study’s conception and design. NF performed the statistical analysis and wrote the first draft of the manuscript. Both authors contributed to the manuscript revision, and read and approved the submitted version of the manuscript.

## Conflict of Interest

The authors declare that the research was conducted in the absence of any commercial or financial relationships that could be construed as a potential conflict of interest.

## Publisher’s Note

All claims expressed in this article are solely those of the authors and do not necessarily represent those of their affiliated organizations, or those of the publisher, the editors and the reviewers. Any product that may be evaluated in this article, or claim that may be made by its manufacturer, is not guaranteed or endorsed by the publisher.

## Abbreviations

ESM: Experience Sampling Method
INCOM: Iowa-Netherlands Comparison Orientation Measure.
